# Empirical approach to the gender gap in students’ reading consumption in international contexts

**DOI:** 10.3389/fpsyg.2023.1304890

**Published:** 2023-12-19

**Authors:** Ester Trigo Ibáñez, Inmaculada Clotilde Santos Díaz

**Affiliations:** ^1^Department of Language Teaching, University of Cádiz, Cádiz, Spain; ^2^Department of Didactics of Languages, Arts and Sports, University of Málaga, Malaga, Spain

**Keywords:** adolescents, co-education, reading habits, types of reading, educational policy

## Abstract

Reading is a very important aspect today, which is why it is a recurring topic in research. This study aims to analyze the gender gap in the reading consumption of adolescents and compare the situation in Spain with that of other countries. It addresses, for the first time, the influence of the gender factor on the reading habits of adolescents who have just begun secondary education. This is an empirical study carried out based on the research projects “Determining factors in the reading habits of secondary education students. A study from the variables of the educational context” and “Reading habits in international contexts of Secondary Education students. A study of educational practices to promote reading.” It is based on the hypothesis that reading consumption is conditioned by gender and country-specific educational policies and, therefore, by the place where the study is carried out. To do this, an international sample of students was taken as a reference, made up of a matrix group of Spanish students contrasted with small samples from three different countries that we are interested in comparing with the Spanish context: Portugal, Poland, and Chile. The results have shown that the gender gap in reading is perceived in all the contexts studied and that it increases in contexts with less developed co-education programs; therefore, it is necessary to implement effective policies in the educational field to eliminate the existence of gender stereotypes.

## 1 Introduction

Reading is an extremely important aspect today. In fact, there is a recurring concern about how literacy processes are developed and, therefore, how the population’s understanding and reading habits are consolidated. Thus, international reports have been published for years, such as PISA, which establishes parameters for measuring educational quality, taking reading literacy as a reference, among other issues (Crato, 2021).

This fact has led to the development of lines of research that address the training of readers from various perspectives, such as the processes of access to the text (Poole, 2005; Cantrell et al., 2017), the emotional dimension present in reading (Jiménez-Pérez et al., 2020; Zaldívar, 2020), the motivation for this activity (Cerezo and Casanova, 2004; Urdan and Schoenfelder, 2006; Hebbecker et al., 2019), and the most conducive teaching strategies for developing readers (Henderson et al., 2020; Casas and López-Pellisa, 2022; Morales and Martín, 2023), to cite a few examples.

In accordance with the above, it is vitally important to know the reading habits of citizens since the possible deficits found will contribute to the programming of effective strategies from both an educational and social point of view. Thus, an entire trend has been developed aimed at knowing these reading habits in Primary Education (Artola et al., 2013, 2017; Pérez-Parejo et al., 2018a,b; Scholes, 2019; Jaraíz et al., 2022; Agrelo-Costas et al., 2023), in Secondary Education (Muñoz and Hernández, 2011; González-Ramírez et al., 2020; Romero-Oliva et al., 2020; Trigo et al., 2020a; González-Ramírez et al., 2022), and in Higher Education (Yubero and Larrañaga, 2015; Pérez Parejo et al., 2019; Johansson, 2021; Escalante et al., 2023). This exploration of reading habits has not only been carried out in the mother tongue, it also transcends to other languages (Pérez-Parejo et al., 2018a; Castillo-Rodríguez and Santos-Díaz, 2022).

The studies have served, among other issues, to establish reading profiles among young people (Jang and Henretty, 2019; Jang et al., 2021; Santos Díaz et al., 2021) and, from the knowledge of these profiles, promote work strategies with schools and public administrations (Trigo et al., 2023).

Within the group of university students, there is a group to which special attention has been paid, namely, future teachers, since they will oversee making true reading and literary mediation effective (González-Ramírez and Pescara, 2023). In this sense, we have studies that explore the beliefs of future teachers about their reading training (Abad Álvarez-Álvarez and Diego-Mantecón, 2019) and works that investigate the reading habits of this group (Castillo-Rodríguez and Santos-Díaz, 2022). However, if it is important to know the reading behavior of future teachers, it is also essential to delve into the reading universe of active teachers. Teachers are, after all, a fundamental piece in the formation of a citizen who becomes a reader. In fact, the school is a very important agent for social change. In this sense, the materials that teachers use in their classes have been analyzed (Ballester Pardo, 2021; Martínez et al., 2021), along with how they bring the school curriculum to the classroom (Estellés, 2013), their own motivation for reading (Griffin and Mindrila, 2023), the strategies they activate to motivate their students to read (Urdan and Schoenfelder, 2006), the type of classroom library they offer their students (Henderson et al., 2020), how they configure reading plans (López de Lérida, 2021; Martínez et al., 2023), and how their beliefs influence the training of readers (Watson et al., 2017).

In this study, we pay special attention to those investigations related to the influence that the gender variable has on the development of reading habits since we consider it an essential aspect of social change (Scheiber et al., 2015). It depends, in part, on the school institution whether gender stereotypes are eradicated (Azúa, 2016). Educational policies gradually contribute to the construction of a more just and equitable society, from early childhood education (Browne, 2001) to higher education (Castillo and Cabezas, 2018), including compulsory schooling (Moreno, 2010).

Therefore, the relationship between gender and reading is a widely studied issue. Broadly speaking, the following seven areas of study were established:

1. Impact of literature on the configuration of gender stereotypes (Gritter et al., 2017; Kneeskern and Reeder, 2020).
2. Relationship between gender and the choice to read over other activities (Chiu and McBride-Chang, 2006; Scholes, 2019).
3. Different reading strategies that boys and girls activate (Poole, 2005; Scheiber et al., 2015);
4. Motivation taking gender as a reference (Cerezo and Casanova, 2004; Marinak and Gambrell, 2010; McGeown et al., 2012).
5. Gender representation in reading materials and plans (López de Lérida, 2021; Martínez et al., 2021, 2023).
6. Gender gap in reading performance (Contreras-Salinas et al., 2020).
7. Reading preferences of students based on gender (Artola et al., 2013, 2017; Agrelo-Costas et al., 2023).

From the analysis of these investigations in general terms, boys declare themselves less motivated by reading than girls, and girls make a more conscious selection of what they read, which is more conditioned by social factors than boys. Boys prefer non-fiction over fiction, and, regarding the most read topics, they prefer adventure, whereas girls are more likely to read romantic literature. Poetry and theater are the genres least likely to be read by young people, who mostly opt for youth novels or non-fiction books, with a more expository nature. It still seems that reading material and plans fail to eliminate gender stereotypes despite having, in the Spanish context, coeducation plans well inserted in the educational field (Angulo et al., 2016) and public policies that favor equality between men and women in Portugal, Poland, and Chile; however, policies in the educational field in these countries are not so evident (Cabanillas, 2015; Flores, 2015; Zawierzeniec, 2022).

For all this, it is necessary to continue investigating the impact of the gender variable on the reading habits of schoolchildren since the results of these investigations will be decisive so that, when transferred to public administrations, they can contribute to changing the situation and, therefore, to eliminating the gender gap that continues to appear among our citizens.

In line with these approaches, in this study, we take as reference an international sample of students made up of a matrix group of Spanish students, which is contrasted with small samples from three different countries that we are interested in comparing with the Spanish context, namely, Portugal, Poland, and Chile. Firstly, Spain’s neighboring country, Portugal, has apparently similar socio-educational conditions but very different results in international reading tests such as PISA (Crato, 2021). Next, Poland is a European country geographically far from Spain and with very marked sociocultural differences (Hernández Carrera et al., 2020). And, finally, Chile, is a geographically remote country but has significant similarities with Spain in terms of its language and culture (Estellés, 2013; Trigo et al., 2022). Although there is existing research that compares the reading habits of different age groups in different countries (Chiu and McBride-Chang, 2006; Yubero and Larrañaga, 2015), this research addresses, for the first time, the gender factor among adolescents who have just begun their secondary education in *ad hoc* selected countries.

Our objective is to analyze the gender gap in the reading consumption of adolescents and compare the situation in Spain with that of other countries. We start from the hypothesis that reading consumption is conditioned by gender and the educational policies of the countries and, therefore, by the place where the study is carried out.

## 2 Method

### 2.1 Participants

This is an empirical study carried out from the “UCA Projects” of the Program to Promote Research Activity (PRIAR) of the University of Cádiz 2017–2018 (PR2017040) “Determining factors in the reading habits of secondary education students. A study from the variables of the educational context” and 2018–2019 (PR2018057) “Reading habits in international contexts of Secondary Education students. A study of educational practices to promote reading.” Researchers from different countries participated in both projects: Spain (University of Cádiz and University of Málaga), Poland (University of Silesia), Portugal (University of Minho), and Chile (Pontificia Universidad Catholic of Valparaíso).

The participants were adolescents in secondary education between the ages of 12 and 13. In Spain, the students were from public centers in seven different provinces: Almería, Barcelona, Cádiz, Cuenca, Granada, Huesca, and Valladolid (*n* = 856). In the rest of the countries, the sample was taken from different countries (*n* = 491): in Chile, specifically in Valparaíso (*n* = 178); in Portugal, in the city of Braga (Portugal) (*n* = 138); and in Poland, in Ustroń and Łódź (*n* = 175).

### 2.2 Instrument

Data collection was carried out by applying a questionnaire prepared by the Ministry of Education, Culture and Sports (hereinafter, MEDC) (The reading habits of Spanish adolescents, 2003) and adapted by Romero-Oliva et al. (2020), with a Cronbach’s Alpha of 0.800. The test was adapted and translated into the mother tongue of the participants when necessary (in Poland and Portugal) and was administered on paper. It consists of 4 initial questions about sociological data (gender, school, grade, and group) and 34 questions related to reading habits and consumption: 28 closed questions on a Likert-type scale and 6 development questions. Some of the questions are made up of dimensions on a specific topic in which the participant must answer more than one item, which facilitates their subsequent analysis as a whole and for each sub-question.

### 2.3 Procedure and analysis

Before applying the questionnaire, the participants’ informed consent was obtained, and it was explained to them that they would be anonymously participating in a research project. To undertake these projects, we had the support of the relevant education authorities in all the contexts explored.

Once the data was collected, each research team entered it into a Google Drive form, which would serve as a preliminary step to create a joint data matrix that would be processed and analyzed with the IBM SPSS Statistics (v.23) statistical package. In this study, three dependent variables are considered and analyzed separately: taste for reading, reading frequency according to genre, and book selection criteria. The independent variables are gender and country (Spain or other country).

The first is composed of a single Likert-type question in which the percentage distribution of each of the categories is shown and the Mann–Whitney *U* test for independent samples is applied, which is a non-parametric test that can be used in ordinal measurement variables. The second and third correspond to two dimensions that consist of various items: the frequency of reading according to genre or type, which is subdivided into 14 items, and the book selection criteria, which is composed of six sections that refer to external criteria and three according to their own criteria.

As with the first variable, the percentage distributions of the subsections are presented, and, subsequently, intersubject factor analysis is carried out for each of the dependent variables. Thanks to the use of more than one factor in the same design, it is possible to analyze the effect of the interaction between the factors (gender and country) at the same time as the two main effects (one for each factor) (Pardo and Ruiz-Díaz, 2005). To this end, it has been proven that the conditions for carrying out this analysis of variance are met: (1) the independent variable is quantitative; (2) the observations are independent of each other; (3) the distribution of the dependent variable is normal; and (4) the variances of the scores of the dependent variable are equal in the two groups. For this, Levene’s *F* test was carried out.

Once the quantitative results were obtained, following the procedure outlined by Cassany and Shafirova (2022), focus groups were held with the students and teachers to provide a deeper understanding of the reasons behind the observed trends.

## 3 Results

### 3.1 Taste for reading

**Figure 1:**
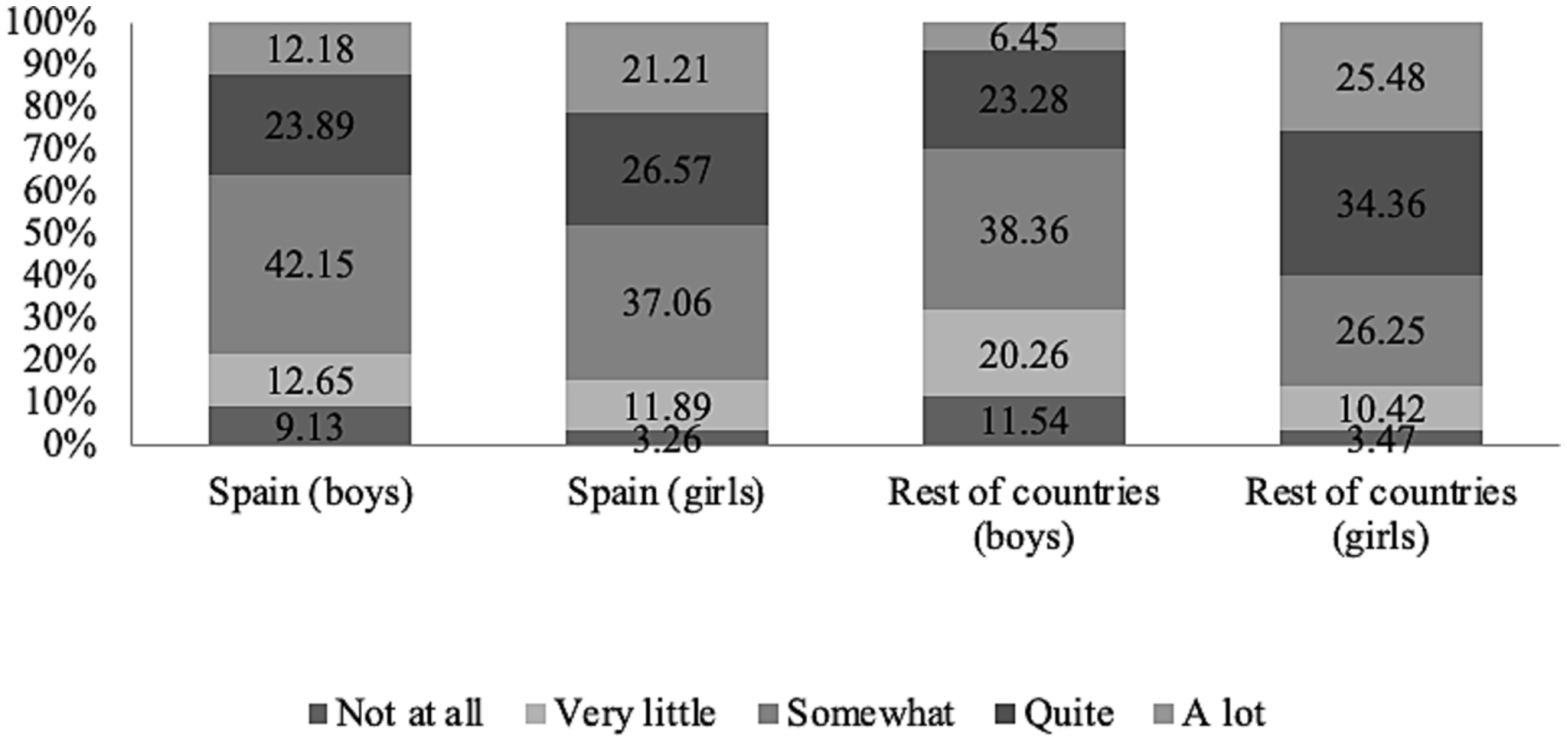
Stacked bar diagram of the assessment of the enjoyment of reading according to sex and geographical area.

To determine their love for reading, the participants were asked the following question: “Do you like to read?.” They were required to choose between five options in response: (1) nothing; (2) very little; (3) something; (4) quite a bit, and (5) a lot. Figure 1 shows the distribution of responses on the different degrees of enjoyment of reading according to gender and geographical area of the study. *A priori*, a considerable difference is observed in certain intervals between girls’ and boys’ sociolect. The average number of boys in Spain who do not like reading at all is 9.13%, while in the case of girls, the percentage drops to 3.26%. On the opposite side, that is, students who said they really like reading, the results are reversed and there is a much higher percentage of girls (21.21%) compared to boys (12.18%). As for the results in the rest of the countries, they are more polarized since the gender gap is even greater. The percentage of boys claiming to not like reading at all is 11.64%, while that of girls is 3.47%, and for those who affirmed that they like reading a lot, the difference is even greater (6.47% of boys compared to 25.48% of girls).

When asked in the focus groups about the tendency of girls to read more than boys, we discovered, in all contexts, that girls associated reading with a more intimate activity and that reading books, especially narrative ones, offered the opportunity to live adventures from their own imagination. The boys, however, in their leisure time, preferred more dynamic activities and the consumption of video games (Table 1).

**Table 1:** Ranks according to gender for the pleasure of reading.

**Ranks**				
Gender		*N*	Average rank	Sum of ranks
Do you like reading?	Boys	659	591,85	390,030,00
	Girls	688	752,69	517,848,00
	Total	1,347		

To reveal the distribution of the data, we performed the Mann–Whitney U test for independent samples. The descriptive data show a lower average rank for boys (591.85) compared to girls (752.69). The null hypothesis establishes that there is no difference in the degree of enjoyment of reading between the two populations (boys and girls), while in the alternative hypothesis, there is. In this case, as Table 2 shows, the value of *p* is less than the significance level, so the null hypothesis is rejected, and it can be concluded that the difference between the population medians is statistically significant (Tables 3, 4).

**Table 2:** Statistical results Mann–Whitney *U* test.

Test statistics^a^	
	Do you like reading?
Mann–Whitney *U* test	172560.000
Wilcoxon *U* test	390030.000
Z	–7.892
Asymptotic sig. (bilateral)	000

^a^Grouping variable: Gender.

**Table 3:** Ranks according to gender for the taste of reading romances.

**Ranks**				
Sex		*N*	Average rank	Sum of ranks
Reading romances	Male	659	483.75	318793.00
	Female	688	856.23	589085.00
	Total	1,347		

**Table 4:** Statistical results Mann–Whitney *U* test.

**Test statistics** ^**a**^	
	Reading romances
Mann–Whitney test	101323.000
Wilcoxon test	318793.000
Z	−18.742
Asymptotic sig. (bilateral)	0.000

^a^Grouping variable: Gender.

### 3.2 Type of reading material

The objective of the next section is to know the reading consumption of adolescents and assess whether there are differences between the types of books that boys and girls read. To do this, the participants answered the question “How much do you read of each of the following types of books?,” choosing between nothing, very little, something, quite a lot, and a lot for each of the 14 types of texts proposed: (1) Mystery/Espionage; (2) Romance; (3) Sports/Health; (4) Adventure; (5) Science-fiction; (6) Terror; (7) Poetry; (8) History/Politics; (9) Humor; (10) Science/Technology; (11) Travel, Nature; (12) Music; (13) Classic literature; (14) Biographies/Autobiographies.

**Figure 2:**
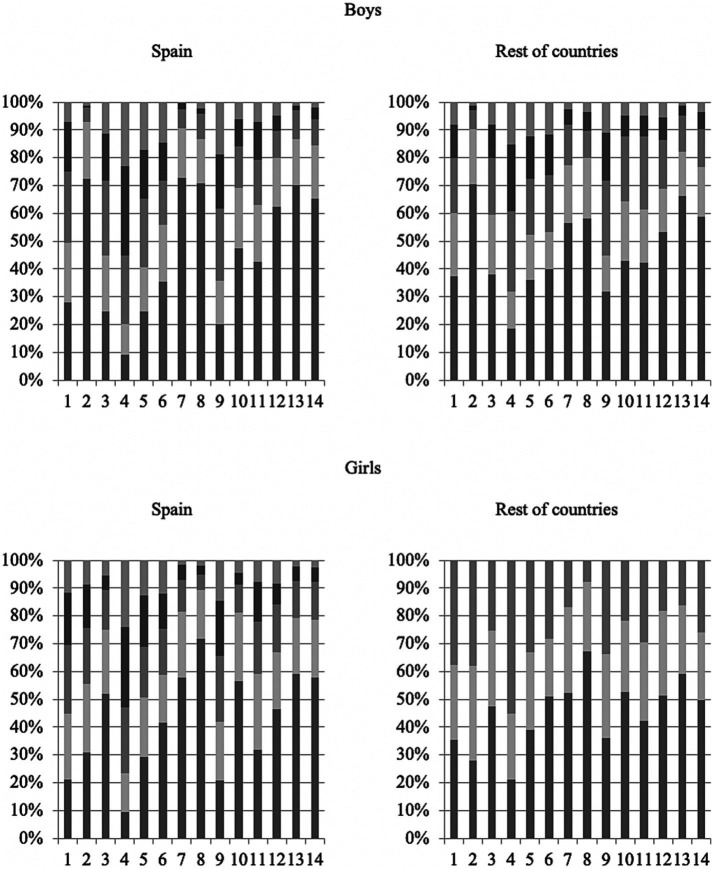
Stacked bar diagram of the percentage of reading frequency by gender according to the type of text divided by sex and geographical distribution.

Figure 2 shows the percentage distribution of the frequency with which they read each type of text according to the study sample analyzed: Spanish boys and girls and those from other countries. In the sample of boys, it is observed that in Spain, the highest percentage of the type of books that they do not usually read at all is poetry (7), at 72.83%, followed by romance (2), at 72.37%, and history or politics (8), at 70.73%. Children from the rest of the countries agreed that romance (2) is the least read in percentage terms, with a percentage of ‘nothing’ responses of 70.55%, followed by classical literature (13), with 66.26%, and biographies/autobiographies (14), with 58.90%.

In the focus groups carried out, the boys stated that they do not usually read poetry because they do not understand its meaning and, furthermore, because it is not a genre that is encouraged in the school environment. On the contrary, they are not usually interested in reading classic literature because they have discovered it through literary study and never through enjoyment. As for romantic literature, they consider it a genre for girls and see it as very far from their interests.

In the entire sample studied, the two types of books that are reportedly never read coincide. Spanish girls claimed to never read about history or politics (8), at 71.79%, followed by classical literature (13), at 59.21%, and poetry (7) and biographies/autobiographies (14), both with 57.81% of responses in that section. On the other hand, girls from the rest of the countries agreed that they never read about history or politics (8), at 61.43%, followed by classical literature (13), at 52.38%, and science and technology (10), at 47.14%.

In the focus groups carried out, the girls alleged the same reasons as the boys for avoiding reading literary classics and poetry. Furthermore, they consider the scientific-technological field to be more related to the masculine world and romantic literature to be more related to the feminine.

As for the types of books reportedly read a lot, Spanish boys and girls have the same top three, albeit with some differences in the percentages. In both cases, the types of reading material that registered the highest number of “a lot” responses are adventure books (4), at 22.95% for boys and 24.01% for girls, followed by humor (9), at 18.74% for boys and 14.45% for girls, and science fiction (5), at 17.10% for boys and 12.8% for girls.

Among the rest of the international students, the highest number of “a lot” responses among Spanish boys was again recorded for adventure books (4), followed by science fiction (5), at 12.27%, and horror (6), at 11.66%. In girls, the highest percentage of “a lot” responses was recorded for romantic books (2), at 24.76%, followed by adventure books (4) and horror books (6), at 17.14%. Therefore, in the four groups analyzed, adventure books occupy the first or second position in terms of the number of participants who affirmed that they read them very frequently.

In the focus groups carried out, in all the contexts explored, a trend was detected in the school environment around promoting the reading of adventure books to satisfy the preferences of boys and girls and, with that, try to alleviate the gender gap.

Overall, the types of books that adolescents from the entire sample affirmed they never read are history or politics (8), at 67.86%, classical literature (13), at 62.82%, and poetry (7), at 60.05%. On the other hand, the types of books with the highest number of responses in the “a lot” category are adventure books (4), at 21.72%, followed by humor books (9), at 15.79%, and science fiction (5), at 14.16%. Although *a priori* the least read types of reading material, such as classical literature and history, are those that are most implicitly inserted in the educational curriculum of the four countries, there are certain books, such as humorous ones, that attract more students’ attention and that they read more frequently, predictably in their leisure time.

It is worth highlighting the disparity in the reading frequency of romantic literature and gender. Boys from Spain and from the rest of the countries said they never read this type of reading material, with percentages of 72.37 and 70.55% respectively, while the percentage for girls is much lower, at 31% in Spain and 16.67% in the rest of the countries. These data are inverted when it comes to the option “quite a lot” and “a lot,” at 0.94 and 1.17% for Spanish boys and 1.84 and 1.23% for boys from other countries. On the other hand, the percentage is much higher in girls, at 15.85 and 8.62% for Spanish girls and 15.24 and 24.76% for girls from the other countries.

Given that, *a priori,* the genre with the greatest percentage difference between boys and girls is romance, a Mann–Whitney *U* test was carried out to check whether said distribution is related to the gender variable. The average range of boys is much lower than that of girls (483.75 versus 856.23) and the asymptotic significance is 0.000, rejecting the null hypothesis that the difference between the medians is the same between the two populations (boys and girls).

Next, a between-subject factor model is carried out to evaluate the main and interaction effects of two independent variables. The effects of the main variables (gender and country) are analyzed, as well as the effect of the interaction between gender and country on reading frequency. For the main effects, we identified the following research questions: “Are there differences between the marginal means in reading frequency depending on whether they are boys or girls?” and “Are there differences in reading frequency depending on the country?.” The analysis of the interaction effects answers the question “Is the effect of male gender different from the effect of female gender depending on the country analyzed?.” There would be no AxB interaction if the reading frequency were the same in both genders.

**Figure 3:**
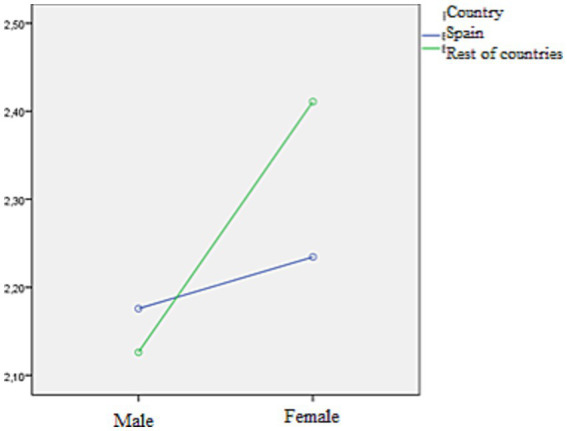
Marginal means of reading frequency according to gender and the sex variable.

Figure 3 represents the cell means. H0 states that there is no AxB interaction, while H1 states that there is an interaction. As can be seen, the lines are not parallel, so the graph suggests a possible interaction. Girls tend to have a higher reading frequency, as is also the case for Spain.

The descriptive statistics in Table 5 show similar means in the population of boys regardless of the country (2.18 in Spain and 2.13 in the rest of the countries), while it is higher in girls, especially in the rest of the countries (2.34 in Spain and 2.41 in the rest of the countries).

**Table 5:** Descriptive statistics of the dependent variable frequency of reading types.

Gender		Mean	Standard deviation	*N*
Boys	Spain	2.1758	0.65247	427
	Rest of countries	2.1262	0.69178	232
	Total	2.1621	0.66334	659
Girls	Spain	2.2344	0.63926	429
	Rest of countries	2.4109	0.66698	259
	Total	2.2924	0.65326	688
Total	Spain	2.2052	0.64617	856
	Rest of countries	2.2865	0.69161	491
	Total	2.2299	0.66107	1,347

Levene’s test (see Table 6) accepts the null hypothesis that the variances are equal since *p* > 0.05; specifically, 0.257, and *F*(1.358). Therefore, the principle of homoscedasticity is met, allowing factor analysis to be applied in a robust way.

**Table 6:** Levene’s equality test of error variances.

*F*	df1	df2	Sig.	
1.358	3	1,225	0.254	
Test the null hypothesis that the error variance of the dependent variable is equal between groups.				

Dependent variable: frequency of different types of reading. ^a^Design: Interception + Gender + Country + Gender * Country.

The results of the between-subject effects test (Table 7) show that the null hypothesis is rejected with *F*(7.633) and *p* < 0.05 and, therefore, there are interaction effects. Given that the main effects are subject to the existence of interaction, the simple effects of each factor (gender and nationality) are analyzed below, keeping each level of another factor constant. In our case, we focus on identifying whether reading frequency is influenced by gender; we therefore segmented the file by country (Spain or other country) and carried out two *T*-student tests for independent samples since the gender variable only has two levels. To check the simple effects of B (in our case gender), we used an alpha of 0.05/number of levels of A (country), which, in this case, is (α = 0.05/2 = 0.025).

**Table 7:** Test of between-subjects effects.

Origen	Type III sum of squares	gl	Mean quadratic	*F*	Sig.	Partial eta squared	Non-centrality parameter	Observed powerbry^b^
Corrected model	9.889^a^	3	3.296	7.666	0.000	0.018	22.998	0.988
Interception	5141.671	1	5141.671	11957.113	0.000	0.907	11957.113	1.000
Gender	7.569	1	7.569	17.603	0.000	0.014	17.603	0.987
Country	1.033	1	1.033	2.403	0.121	0.002	2.403	0.341
Gender * Country	3.282	1	3.282	7.633	0.006	0.006	7.633	0.788
Error	526.761	1,225	0.430					
Total	6647.587	1,229						
Corrected total	536.651	1,228						

Dependent variable: frequency of different types of reading. ^a^*R* squared = 0.018 (adjusted *R* squared = 0.016). ^b^It has been calculated using alpha = 0.05.

In Spain, the average sociolect for boys and girls is very similar (2.17 in boys and 2.34 in girls), while in the rest of the countries, it is higher (2.12 in boys and 2.41 in girls). Both in Spain and in the rest of the countries, Levene’s *F* statistic assumes that the variances are equal. In the Spanish sample, the null hypothesis of equality of means is accepted (*p* > 0.025), while in the rest of the countries, the null hypothesis is rejected, and it is shown that there are differences between the means of boys and girls according to reading frequency (Tables 8, 9).

**Table 8:** Test of independent samples in the Spanish population.

		Levene’s test for quality of variances		*t*-test for equality of Means						
		*F*	Sig.	*t*	gl	Sig. (bilateral)	Means Difference Standard	Error difference	95% Confidence Interval of the difference	
									Lower	Upper
Frequency of different types of reading		0.719	0.397	−1.328	854	0.185	−0.05862	0.04415	−0.14528	0.02804
	No Equal variances are not assumed			−1.328	853.461	0.185	−0.05862	0.04415	−0.14528	0.02804

Country = Spain.

**Table 9:** Independent sample testing in the foreign population.

		Levene’s test for quality of variances	t-test for equality of Means							
		*F*	Sig.	*t*	gl	Sig. (bilateral)	Means difference	Standard error difference	95% de confidence interval of the difference	
									Lower	Upper
Frequency of different types of reading	Equal variances are assumed	0.893	0.345	−4.023	371	0.000	−0.28468	0.07077	−0.42383	−0.14553
	Equal variances are not assumed			−4.004	342.076	0.000	−0.28468	0.07109	−0.42451	−0.14484

Country = Rest of countries.

#### 3.2.1 Book selection criteria

This dimension aims to reveal the most frequent criteria that adolescents use when choosing the books they read and whether there is a relationship between gender and country with respect to the variable dependent on external criteria. To do this, the participants answered the question, “How often do you use the following criteria to select the books you read?,” choosing between the following options: never, almost never, sometimes, frequently, very frequently. The dimension is divided into nine criteria, of which, the first six are considered external since they depend on other agents and the last three on something that catches the reader’s attention: (1) recommended by friends; (2) recommended by teacher; (3) recommended by family; (4) received as a gift; (5) already available at home; (6) fashion or advertising; (7) attracted to the topic; (8) attracted to the author, and (9) attracted to the cover.

The results show that the percentage of “never” responses regarding the selection of criteria in boys is much higher than that of girls, both in Spain and in the rest of the countries. In Spanish boys, the highest percentage of “never” responses was recorded for fashion or advertising (6), followed by attracted to the author (8), at 39.6%, and recommended by friends (1), at 26.23%. In children from other countries, the fashion or advertising factor again received the most “never” responses (6), at 46.01%, followed by recommended by teacher (2), at 37.42%, and attracted to the author (8), at 31.90%.

Among Spanish girls, the criterion with the most “never” responses is attracted to the author (8), at 39.6%, followed by fashion or advertising (6), at 32.6%, and attracted to the cover, at 16.8%. In girls from other countries, the criterion of recommended by teacher (2) is used the least, at 27.60%, followed by recommended by family (3), at 20.95%, and attracted to the author (8), with a percentage of 20.48%.

The criteria that the Spanish participants claimed to use very frequently coincide in girls and boys: attracted to the topic (7), at 38% in boys and girls; received as a gift (4), at 23.19% for boys and 23.31% for girls; already available at home (5), at 19.70% for boys and 17.72% for girls; and recommended by family (3), at 10.77% for boys and 15.38% for girls. Therefore, more than the effect of school, the importance of books being recommended or available at home is highlighted.

In the focus groups carried out in the Spanish context, it is confirmed that the topic is the most important aspect for boys and girls and there is little influence from the school environment when it comes to selecting a book that they think they will enjoy reading. Boys and girls confirm the influence of their peers when selecting their reading material.

As for the sample from other countries, the most common criteria for choosing books change depending on the sex of the informants. In boys there is a large percentage difference with respect to girls in the “very frequently” option since they have marked it on average at 18.73% compared to girls who do so at 9.08%. Therefore, it seems that boys are less clear about the criteria they use to select books. The criterion that boys most often chose is attracted to the topic (7), at 26.38%, followed by attracted to the cover, at 12.30%, and attracted to the author, at 9.82%. With respect to girls, the criterion recorded with the highest percentage of “very frequent” responses is attracted to the topic (7), at 53.33%, followed by attracted to the author (8), at 22.86%, and received as a gift (4), at 22.86%.

**Figure 4:**
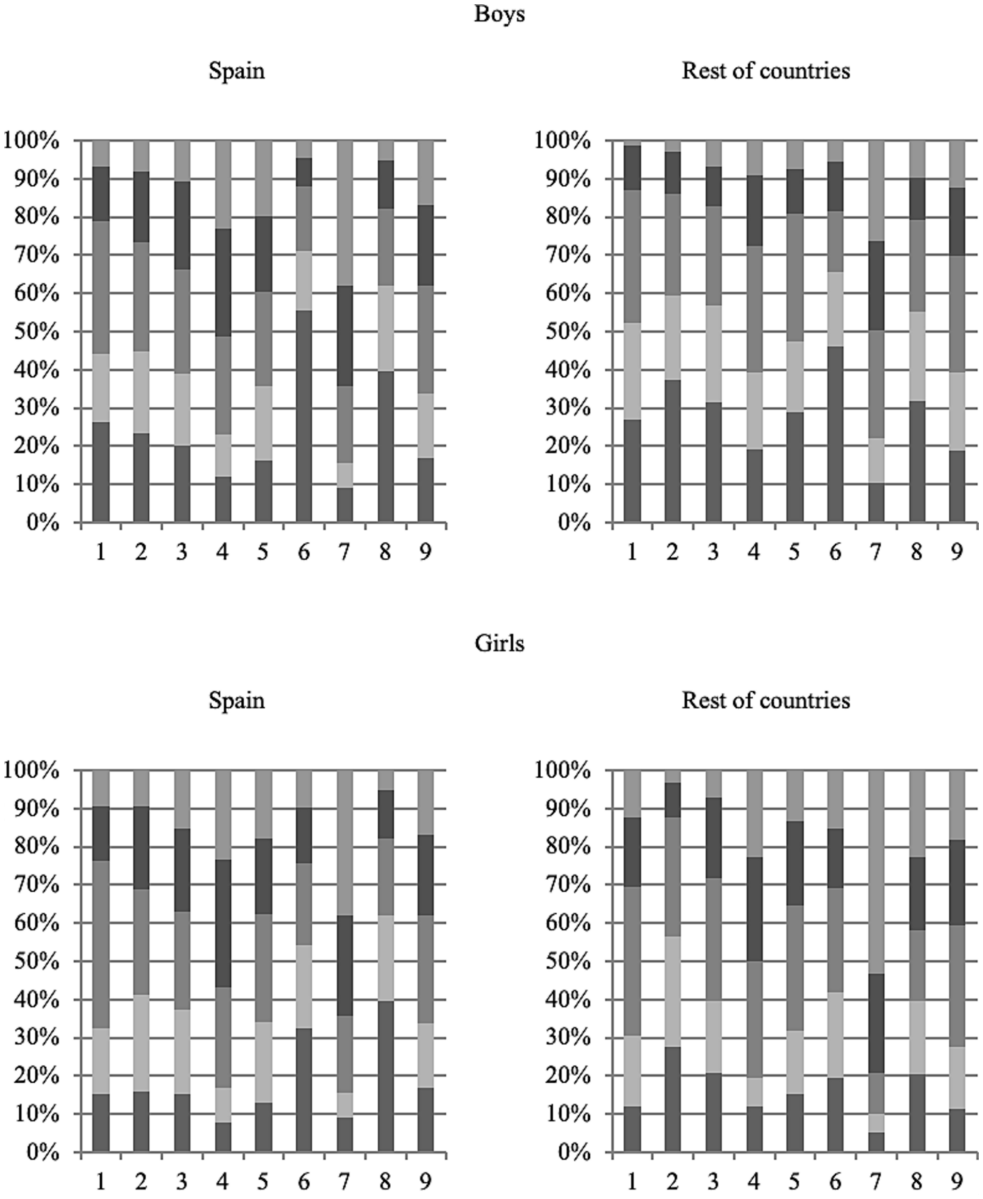
Stacked bar diagram on the criteria for choosing a book according to sex and geographical distribution.

Once again, in the international contexts explored, both boys and girls indicate the topic as the most attractive reason for selecting a book and, once again, there is little influence of teachers as mediators in their selection of reading material (Figure 4).

The criteria in which there is the greatest disparity in responses is “fashion or advertising” with respect to gender and the country analyzed; boys tend to follow fashion less and in Spain, they follow fashion less than in the rest of the countries. A total of 70.96% of Spanish boys marked the “never” or “almost never” options with respect to this criterion; in Spanish girls, the percentage drops to 54.31%; in boys from other countries, it is 65.44%; and in girls from other countries, it is 41.90%. When performing the Mann–Whitney *U* test between gender and this criterion, the null hypothesis is rejected because the value of p is less than the level of significance, so the difference between the medians is not the same between the two populations (boys and girls).

Next, a 2 × 2 analysis of variance was carried out with the factors gender (boys and girls) and country (Spain or other country). The dependent variable was the external selection criterion when choosing a book, coming from a dimension composed of six sub-criteria. Levene’s equality test tests the null hypothesis that the error variance of the dependent variable is equal between groups (*F* = 1.657 and *p* = 0.174). The descriptive statistics in Table 10 show that girls’ sociolect means are higher, indicating that they allow themselves to be guided more by external factors when choosing a book. Likewise, this average is higher in the case of Spain compared to other countries. Regarding the interaction between the factors of gender and country, it was statistically significant, with *F* = 10.046 and *p* < 0.05 (see Table 11).

**Table 10:** Descriptive statistics of the dependent variable external selection criteria.

Gender		Mean	Standard deviation	*N*
Boys	Spain	2.7432	0.73514	427
	Rest of countries	2.3814	0.77750	163
	Total	2.6432	0.76377	590
Girls	Spain	2.9650	0.68240	429
	Rest of countries	2.8905	0.75345	210
	Total	2.9405	0.70682	639
Total	Spain	2.8544	0.71742	856
	Rest of countries	2.6680	0.80382	373
	Total	2.7978	0.74929	1,229

**Table 11:** Test of between-subjects effects.

Origen	Type III Sum of Squares	gl	Mean Quadratic	*F*	Sig.	Partial eta squared	Non-centrality Parameter	Observed Powerbry^b^
Corrected model	43.340^a^	3	14.447	27.390	0.000	0.063	82.171	1.000
Interception	7743.323	1	7743.323	14681.075	0.000	0.923	14681.075	1.000
Gender	34.316	1	34.316	65.062	0.000	0.050	65.062	1.000
Country	12.228	1	12.228	23.184	0.000	0.019	23.184	0.998
Gender * Country	5.298	1	5.298	10.046	0.002	0.008	10.046	0.886
Error	646.109	1,225	0.527					
Total	10309.694	1,229						
Corrected total	689.449	1,228						

Dependent variable: external selection criteria. ^a^*R* squared = 0.063 (*R* squared adjusted = 0.061). ^b^Calculated using alpha = 0.05.

Given that the interaction is significant, we proceed to decompose the factorial design into a set of simple designs, which in this case, consists of knowing the difference between means according to gender in the population of Spain, on the one hand, and in the population of the rest of the countries, on the other hand. To do this, we carried out two student T-tests for independent samples. Thus, we segmented the sample according to country and calculated and applied an alpha α = 0.05/2 = 0.025. The results show that there is equality between the variances in both tests and that the significance is positive in both populations, therefore rejecting the null hypothesis of equality of means (see Tables 12, 13). This means that girls from both Spain and other countries tend to use external factors more frequently when selecting a book.

**Table 12:** Test of independent samples in the Spanish population.

		Levene’s test for quality of variances		t-test for equality of Means						
		*F*	Sig.	*t*	gl	Sig. (bilateral)	Means difference	Standard error difference	95% de confidence interval of the difference	
									Lower	Upper
Frequency of different types of reading.	Equal variances are assumed	0.686	0.408	−4.576	854	0.000	−0.22187	0.04848	−0.31702	−0.12671
	No equal variances are assumed			−4.576	848.708	0.000	−0.22187	0.04849	−0.31704	−0.12669

Country = Spain.

**Table 13:** Independent sample testing in the foreign population.

		Levene’s test for quality of variances		*t*-test for equality of Means						
		*F*	Sig.	*t*	gl	Sig. (bilateral)	Means difference	Standard error difference	95% de confidence interval of the difference	
									Lower	Upper
Frequency of different types of reading	Equal variances are assumed	0.067	0.797	−6.383	371	0.000	−0.50909	0.07976	−0.66592	−0.35225
	No Equal variances are assumed			−6.358	342.989	0.000	−0.50909	0.08007	−0.66658	−0.35159

Country = Rest of countries.

## 4 Discussion

Throughout this study, we have sought to understand the behavior of adolescents in terms of three dimensions related to reading consumption: the degree of love for reading, the type of reading material they usually read, and the criteria most used when selecting a book. Likewise, gender and country were taken as independent variables, distinguishing between the sample taken in different parts of Spain and that of other countries (Chile, Poland, and Portugal). In previous works, we assessed the analog reading consumption of adolescents (Trigo et al., 2020a) and the reading preferences of young people regarding the medium: digital or paper (Santos-Díaz et al., 2023).

In this study, however, our main objective is to know the gap between the reading consumption of adolescents according to gender and to compare the situation in Spain with that of other countries. We started from the hypothesis that reading consumption is conditioned by gender and the educational policies of the countries and, therefore, by the place where the study was carried out. The results of our work confirm this hypothesis since there still seem to be opportunities for improvement in this area (Ballester Pardo, 2021; López de Lérida, 2021, among others), although there seems to be a positive influence of the coeducation policies carried out in Spain (Angulo et al., 2016).

This is a novel investigation as it contrasts the results of a large Spanish sample with three very significant and well-differentiated countries: Portugal, due to geographical proximity, Poland, due to geographical distance, and Chile, due to cultural proximity. The results obtained may serve as a basis for working, in the contexts studied, on the initial and ongoing training of teachers, providing teachers in training and in practice with appropriate strategies to exercise effective reading and literary mediation.

In general, our results show that girls tend to express greater pleasure in reading, a greater frequency of reading, and even a stronger predisposition to book selection criteria (internal or external) than boys. These results coincide with those of previous research (Cerezo and Casanova, 2004; Marinak and Gambrell, 2010; Watson et al., 2017). The findings relating to this gender gap are striking considering that, from an early age, efforts have been made to provide equal reading education to boys and girls. To do this, practices such as reading aloud have been used (Cova, 2004; Trigo et al., 2020b).

With respect to the first dimension analyzed, there is a much higher percentage of girls who say that they really like to read compared to boys (21.21% compared to 12.18%). The Mann–Whitney U test indicates that there are differences between the means, and, above all, this difference becomes more latent in other countries compared to Spain (González-Ramírez et al., 2022). This corroborates the results of previously carried out research that shows a predisposition among children to read non-fiction rather than literary reading (Gritter et al., 2017; Malloy et al., 2017; Alexander and Jarman, 2018; Kneeskern and Reeder, 2020).

In dimension 2, it has been shown through a factor analysis that children from other countries tend to read the least, regardless of gender. Furthermore, there are significant differences between the reading frequency of boys and girls from other countries that do not occur in the Spanish sample, where the recorded means are more similar in the two genders. As for the types of reading, adventure books registered a greater frequency of reading in all populations, unlike other genres that are more frequent in girls than in boys, such as romance (Munson-Warnken, 2017; González-Ramírez et al., 2020; Trigo et al., 2020a).

Added to the above is boys’ predilection for reading in digital environments compared to girls’ desire to read on paper, an issue that has been demonstrated in recent research. It would be interesting, in future research, to analyze in greater depth the incidence of the gender variable in the choice of reading medium since, until now, previous studies have only found a correlation between a lack of reading habits and the choice of digital media (Loh and Sun, 2019; Jang et al., 2021; Santos-Díaz et al., 2023); however, it remains to be determined whether gender is a variable that should be considered, as this would allow effective decisions to be made regarding teaching methods (Urdan and Schoenfelder, 2006; Scholes, 2019; Martínez et al., 2021).

Next, in dimension 3, the most frequently used criteria when selecting a book are presented. The 2×2 analysis of variance shows that there is an interaction between the factors of gender and country. Regarding the main effects, the differences between means with gender are significant both in the Spanish sample and in the samples from the rest of the countries, so girls tend to be guided more frequently by external factors when choosing a book than boys (Artola et al., 2017; Griffin and Mindrila, 2023). Regarding the results by subsections, the most recurrent criterion when selecting a book is “attracted to the topic,” but the criteria “recommended by friends” and “recommended by family” are also commonly selected before” recommended by teacher.” These results coincide with previous research (Trigo et al., 2020a; González-Ramírez et al., 2022).

Furthermore, to help us with the interpretation of the data, we held two focus groups. One group was with students, where they were urged to express their reading preferences and to mention reading practices at the educational center. The other focus group was made up of teachers, who discussed not only the reading consumption of young people but also the selection of books and strategies to promote reading, where organizational aspects of the center and related to educational policies came to light, and the revitalization of the school library and the co-education program. However, it seems that boys and girls are not influenced by the mediation efforts made by the school and continue to rely on criteria external to the school to select their reading material.

In line with these results, although the gap between genders is greater in other countries compared to Spain, it should be noted that it is latent in one way or another in the three dimensions analyzed (Contreras-Salinas et al., 2020). Spanish girls tend to show a greater taste for reading and have a higher tendency to take into consideration criteria for selecting books, both external and their own. At the same time, it is striking that, even with co-education policies, certain types of reading material are more frequent in girls, such as romantic literature, an issue that leaves space for reflection since it seems that the efforts that have been carried out in this regard are not working adequately (Browne, 2001; Araya-Umaña, 2004; Moreno, 2010) and very marked gender stereotypes continue to be found (Azúa, 2016).

As a limitation of the study, it should be mentioned that it would have been interesting to have the participation of students from more different countries. In this sense, an opportunity for a new line of research is presented in which we could analyze not only the incidence of the gender gap but also how specific educational policies influence this gender gap. Having used a validated questionnaire and a representative sample in Spain, this study could serve as a reference for future comparisons. Moreover, we have confirmed that the results achieved are in line with those of previous research and could serve as an excellent starting point for the research community to reflect on. We have managed to demonstrate that the gender gap in reading is perceived in all the contexts studied and that it increases in contexts with less developed co-education programs; therefore, it is necessary to implement effective policies, in the educational field, to eliminate the existence of gender stereotypes, an issue that must be addressed in future research.

## Data availability statement

The raw data supporting the conclusions of this article will be made available by the authors, without undue reservation.

## Ethics statement

Ethical review and approval was not required for the study on human participants in accordance with the local legislation and institutional requirements. Written informed consent for participation in this study was provided by the participants’ legal guardians/next of kin.

## Author contributions

ET: Conceptualization, Formal analysis, Funding acquisition, Methodology, Project administration, Software, Validation, Writing – original draft, Writing – review & editing. IS: Data curation, Formal analysis, Methodology, Writing – original draft, Writing – review & editing.
